# Thermal Stress Has Size‐Dependent but Not Sex‐Specific Effects on Mortality in an Insect Model

**DOI:** 10.1002/ece3.74020

**Published:** 2026-07-12

**Authors:** Isobel Grieve, Natalie Pilakouta

**Affiliations:** ^1^ Centre for Biological Diversity, School of Biology University of St Andrews St Andrews UK

**Keywords:** burying beetles, climate change, survival, temperature

## Abstract

Climate change is raising average temperatures and intensifying the frequency and severity of heatwaves, threatening animal populations. Ectotherms are especially vulnerable to increased temperature due to their limited capacity for thermoregulation, with studies indicating reduced survival under heat stress. Within‐population variation in mortality under stressful temperatures could limit our ability to make broader ecological predictions. For example, males and females can exhibit physiological and behavioural differences that extend to differences in heat sensitivity. Body size may also influence temperature tolerance and could contribute to such differences. To this end, we exposed male and female burying beetles (*Nicrophrous vespilloides*) of a wide range of body sizes to a simulated heatwave of 26°C and examined the resulting mortality rates compared to control beetles kept at 20°C. We found that beetles exposed to the heatwave suffered significantly increased mortality, but there was no difference between males and females. We also found that a smaller body size was associated with significantly higher mortality following heatwave exposure. This size‐dependent effect suggests that warming‐induced reductions in body size may also indirectly result in higher mortality rates, thereby exacerbating the effects of climate change on animal populations. Overall, our findings highlight the importance of considering sources of within‐population heterogeneity when measuring responses to climate change, as traits such as body size may mediate thermal sensitivity even in the absence of sex‐specific differences.

## Graphical abstract

Climate change is increasing the frequency and intensity of heatwaves, posing a major risk to animal populations. We exposed male and female burying beetles of a wide range of body sizes to a simulated heatwave and found that heat stress increased mortality. Smaller individuals were especially vulnerable, but mortality did not differ between the sexes. These findings highlight the importance of considering intraspecific variation when predicting insect responses to warming.

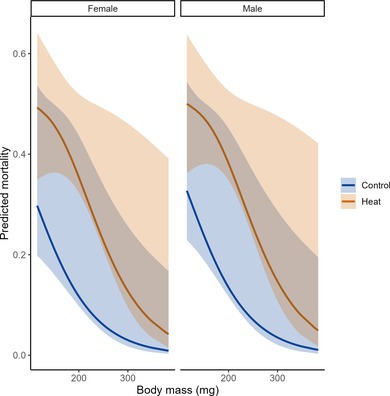

## 1 Introduction

Anthropogenic climate change is a pervasive and growing threat for global ecosystems (Nunez et al. 2019; Pörtner et al. 2022; Heinen et al. 2025). The current rate of biodiversity loss is congruent with the past five mass extinctions (Rull 2021), driven in part by rising global mean temperatures and an increased frequency and intensity of heatwaves (Meehl and Tebaldi 2004; Urban 2015; Luo et al. 2024). The effects of climate change may be especially pronounced for ectotherms, due to their limited capacity for thermoregulation (Deutsch et al. 2008; Hayden Bofill and Blom 2024). Across a diverse range of insect taxa, elevated temperatures consistently reduce both juvenile and adult survival and impact key life history traits (e.g., Thomas et al. 2004; Kingsolver et al. 2016; González‐Tokman et al. 2020; Harvey et al. 2023). For instance, adult leafminer moths (*Tuta absoluta*) exposed to intense heat stress showed significantly increased mortality and life‐stage specific sensitivity of longevity under temperature stress (Zhou et al. 2024). A 5°C increase in ambient temperature reduced survival in dragonfly larvae (*Erythemis collocate*; McCauley et al. 2018), and temperature stress during rearing resulted in a sixfold increase in mortality rate for *Pieris napi* butterfly pupae (Moradinour et al. 2023). A compilation of thermal tolerance estimates found that across latitude and elevation, most insects do not have sufficient physiological safety margins to survive projected temperatures under climate change (Sunday et al. 2014).

Nevertheless, the usefulness of these estimates for predicting population persistence under climate change depends on their consistency across contexts. Within‐species heterogeneity could limit the capacity for generating broader ecological estimates. For example, males and females can exhibit differences in a range of physiological and behavioural traits, such as life history strategies (Tarka et al. 2018), sexual size dimorphism (Stillwell et al. 2010), senescence (Clutton‐Brock and Isvaran 2007) and geographic ranges (Gissi et al. 2023), with implications for sex‐specific thermal tolerance. The Darwin–Bateman paradigm (Bateman 1948; Dewsbury 2005), with later elaboration from Trivers (1972), proposed that divergent reproductive investment between males and females could generate fundamental differences in optimal mating rates and parental investment, leading to counteracting sources of selection and sex‐specific trait optima. Such theoretical predictions typically lead to the assumption that males exhibit a faster pace‐of‐life and invest more into reproduction than survival or self‐maintenance (Tarka et al. 2018). However, Slatkin (1984) later suggested that the exploitation of different ecological niches and competition over limited resources could generate disruptive sex‐specific selection independent from gametic differences (De Lisle 2019; Mokos et al. 2021).

A growing body of literature has indicated that such sex differences could extend to heat sensitivity and indeed, mortality under temperature stress. Male flour beetles ( *Tribolium castaneum* ) showed greater survival than females under a 5‐day simulated heatwave (Sales et al. 2021), and male *Drosophila melanogaster* produced more heat‐shock proteins at high temperatures compared to females, resulting in increased longevity (Sørensen et al. 2007). Yet, an elevated rearing temperature in the copper butterfly (*Lycaena tityrus*) resulted in greater heat‐shock protein expression in females but not males (Karl et al. 2009); females had significantly higher thermal tolerance across six *Daphnia* populations (Vey et al. 2026); and female adult yellow dung flies tolerated a stressful temperature for significantly longer than adult males (Blanckenhorn et al. 2014). In a comparative study of 11 closely related *Drosophila* species, females were more heat resistant than males in five species, while males showed greater resistance in only two; the other four species displayed similar thermal tolerances between sexes (Mitchell and Hoffmann 2010). A meta‐analysis of ectothermic thermal acclimation capacity across the sexes also found that both males and females were similar in heat tolerance plasticity, although these findings were heterogenous across contexts (Pottier et al. 2021). Nevertheless, over 75% of the estimates included in this synthesis failed to report the sex or confounded both sexes (Pottier et al. 2021). Making generalised predictions of sex differences in response to climate change therefore remains difficult (Gissi et al. 2023; Dougherty et al. 2024; Chatten et al. 2025).

Body size is another fundamental source of within‐species variation that may have a strong influence on thermal tolerance (Stevenson 1985; Peters 1986). Smaller individuals typically have lower energy resources, and a larger surface‐to‐volume ratio, which can determine the rate of heat exchange (Rall et al. 2012; Sentis et al. 2024). In a heat‐transfer model, larger ectothermic body masses permitted a broader body temperature range than small body masses (Stevenson 1985). Empirically, larger body sizes have been linked with increased survival in marine fish species (Thresher et al. 2007; Lorenzen 2022), lizards (Chamaillé‐Jammes et al. 2006) and insects (Reim et al. 2006). Body size may also contribute to sex‐specific heat sensitivity, with sex differences in acclimation capacity associated with differences in body mass between females and males (Pottier et al. 2021). Indeed, sexual size dimorphism determined sex‐specific mortality in the great skua *Stercorarius skua* (Kalmbach et al. 2005), the great tit *Parus major* (Oddie 2000) and the black‐billed magpie *Pica pica* (Lee et al. 2010). However, this relationship between sexual size dimorphism and sex‐specific mortality has mainly been studied in birds, and its relevance for other taxa remains unclear. The evolution of sexual size dimorphism in insects is often attributed to the Darwin–Bateman paradigm, with males emerging faster to maximise mating opportunities, reducing their development period and subsequent body size (Tarka et al. 2018). Differences in body size between the sexes could therefore have both independent effects on variation in mortality under temperature stress and may contribute to sex‐specific differences.

To better understand what might drive intraspecific variation in mortality under heat stress, we investigated the potential roles of sex and body size, using the burying beetle *Nicrophorus vespilloides* as a model system. Burying beetles are well‐suited for investigating intraspecific variation as thermal responses in this species are highly heterogenous within populations. For instance, previous work has demonstrated substantial variation in reproductive outcomes depending on both the timing of heatwaves (Pilakouta et al. 2023) and the type of thermal stress experienced (Pilakouta et al. 2024). Responses to heat stress may also vary across life stages (e.g., Sidhu et al. 2024; Grieve and Pilakouta 2026; Wiil and Pilakouta 2026), breeding bouts (Wiil et al. 2026) and breeding strategies (Sun et al. 2014). Body size is an important component of burying beetle life history (Smith et al. 2026) shown to impact nutritional stress responses in a related species (Trumbo and Xhihani 2015), but this has not been investigated in relation to thermal stress. There is also evidence for sex‐specific effects of simulated heatwaves on reproductive success in this species, with female fertility being more sensitive to heat stress than male fertility (Grieve and Pilakouta 2026).

Here, we examined the effect of ecologically relevant heat exposure on mortality in male and female burying beetles of a wide range of body sizes. We expected heat stress to reduce overall survival, with more pronounced effects in females, considering prior work on reproduction in this species (Grieve and Pilakouta 2026). We also predicted increased mortality in individuals with smaller body sizes as small individuals may overheat more easily (e.g., Reim et al. 2006; Aragón and Fitze 2014; Avilés‐Hernández et al. 2025). Given the consequences of sex‐specific mortality under temperature stress for population sex ratios and mating opportunities, and the ecological significance of body size for life history and population dynamics, understanding such intraspecific variation in thermal responses is of vital importance.

## 2 Methods

### 2.1 Study Species

*Nicrophorus vespilloides* is a carrion beetle that breeds on small vertebrate carcasses and exhibits facultative biparental care. When a mating pair of beetles locate a carcass, they prepare it for reproduction by burying it, stripping the carcass of fur or feathers, shaping it into a brood ball and smearing the surface with antimicrobial secretions. Burial may protect the carcass and brood from competitors and environmental stressors, such as elevated temperatures (Trumbo 2023). Rounding the carcass and removing fur or feathers could increase the amount of flesh accessible for larval consumption and facilitate the application of anal and oral antimicrobial secretions (Potticary et al. 2024). These exudates contain antimicrobial compounds, including lysozymes, which inhibit bacterial and fungal growth and reduce microbial competition (Cotter and Kilner 2010; Arce et al. 2012; Shukla et al. 2018). During carcass preparation, males and females mate repeatedly, with the female laying eggs in the surrounding soil approximately 24–48 h after carcass deposition. Upon hatching, larvae crawl into a crypt or cavity in the carcass where they both self‐feed and are provisioned with pre‐digested carrion by their parents. Larvae disperse from the carcass approximately 1 week after hatching to pupate in the soil, eclosing around 3 weeks later. Under controlled laboratory conditions, individuals reach sexual maturity 10 days post‐eclosion and generally do not survive beyond 65 days (Cope et al. 2021).

### 2.2 Animal Husbandry

We used first‐ and second‐generation laboratory‐bred beetles maintained at the University of St Andrews. The population originated from wild‐caught beetles collected at Bonnytown Farm and Tentsmuir Nature Reserve in Fife, Scotland in August and September 2024. Adults were stored individually in transparent plastic containers (12 × 8 × 2 cm) filled with 1 cm of moist soil and kept at 20°C in cooled incubators (LMS Series IA Model 280 NP) under complete darkness to mimic subterranean conditions. Relative humidity was maintained by keeping the soil moist, and beetles were fed cubes of organic beef *ad libitum* twice a week.

### 2.3 Experimental Design

Experimental beetles were obtained from stock populations as newly‐eclosed adults. Males and females were weighed using a precision balance (Ohaus Pioneer) before being randomly assigned into two treatments: a control treatment where beetles were kept at 20°C for 72 h, and a heat stress treatment where beetles were exposed to a 72 h simulated heatwave of 26°C before being placed back into the control incubator (20°C) for subsequent monitoring. A heatwave of 26°C for 72 h has been shown to be detrimental for reproduction (Pilakouta et al. 2023, 2024; Grieve and Pilakouta 2026) and larval survival in this species (Wiil and Pilakouta 2026), but its effects on adult mortality are unstudied. The intensity and duration of the heatwave were indicative of typical heatwaves in central and south‐eastern Scotland to improve ecological relevance (O’Neill and Tett 2019). Beetles were maintained in two incubators set to constant temperatures (20°C or 26°C).

Individual beetle survival was assessed at the end of the simulated heatwave (72 h from the start of exposure) and every 3–4 days thereafter for 2 weeks. These repeated assessments allowed us to estimate both immediate and short‐term mortality, a period during which differences in survival due to heat stress are expected to be most pronounced (e.g., Melone et al. 2024). The sample sizes were as follows: *n* = 83 males in the control treatment, *n* = 83 females in the control treatment, *n* = 94 males exposed to the simulated heatwave, and *n* = 95 females exposed to the heatwave.

### 2.4 Statistical Analysis

All statistical analyses were conducted in R (version 4.5.1). We used generalised linear mixed‐effects models (GLMMs) or linear mixed‐effects models (LMMs) from the ‘lme4’ package (Bates et al. 2015) to account for variance introduced by using different laboratory generations. Overall mortality was analysed using a GLMM fitted with a binomial error distribution, with treatment, sex and mass included as fixed effects and generation included as a random effect. Interaction terms were not significant and were thus excluded from the final model to avoid overfitting. We also tested for differences in average body mass between males and females, using a linear mixed effects model with sex as a fixed effect and generation as a random effect.

To examine variation in the timing of mortality (i.e., longevity) during the experiment, we fitted a Cox proportional hazards regression model using the ‘survival’ package (Therneau 2026), which controls for uneven intervals and right censoring. Mortality at each observation interval was modelled against treatment and sex, with the generation included as a random effect using the ‘frailtypack’ package (Rondeau et al. 2019). For all models, significance of the fixed effects was assessed using Type II Wald chi‐square tests using the ‘Anova’ function from the ‘car’ package (Fox and Weisberg 2019). Model assumptions and fit were evaluated using simulation‐based residual diagnostics implemented in the DHARMa package (Hartig 2026).

## 3 Results

**FIGURE 1:**
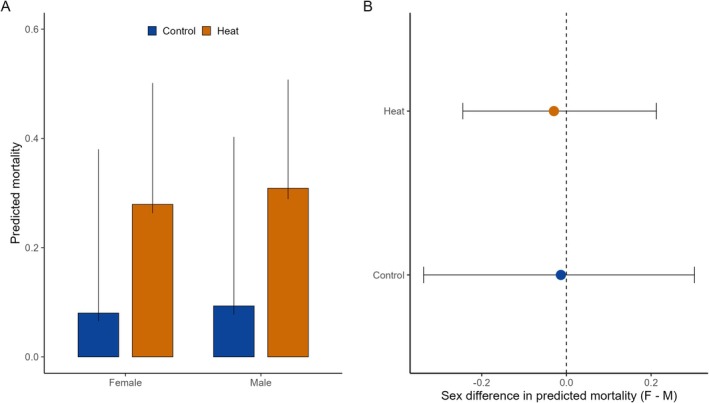
Predicted mortality by treatment and sex, showing (A) model‐predicted mortality probabilities (±95% confidence intervals) for males and females under the control treatment (blue) and heatwave treatment (orange), estimated from a binomial generalised linear mixed‐effects model. (B) Difference in predicted mortality between females and males (female−male) for each treatment. Points represent model‐predicted differences and error bars are 95% confidence intervals. The dashed line indicates no sex difference in mortality.

Beetles exposed to a heatwave had significantly higher mortality compared to those in the control treatment (LRχ^2^ = 13.19, df = 1, *p* < 0.001, Figure 1, Table 1). Contrary to our prediction, there was no difference in mortality between the sexes (LRχ^2^ = 0.21, df = 1, *p* = 0.65).

**TABLE 1:** Proportion and percent (%) mortality, mean survival (longevity in days) and body mass (mg) (±standard deviations) by treatment and sex.

	Control		Heat	
	Male	Female	Male	Female
Mortality *n* (%)	6/83 (7.2%)	2/83 (2.4%)	15/94 (16.0%)	15/95 (15.8%)
Survival (days) (mean ± SD)	13.2 (±2.8)	13.8 (± 1.5)	12.5 (±3.5)	12.6 (±3.5)
Body mass (mg) (mean ± SD)	223 (±50)	235 (± 47)	230 (±40)	234 (±43)

**FIGURE 2:**
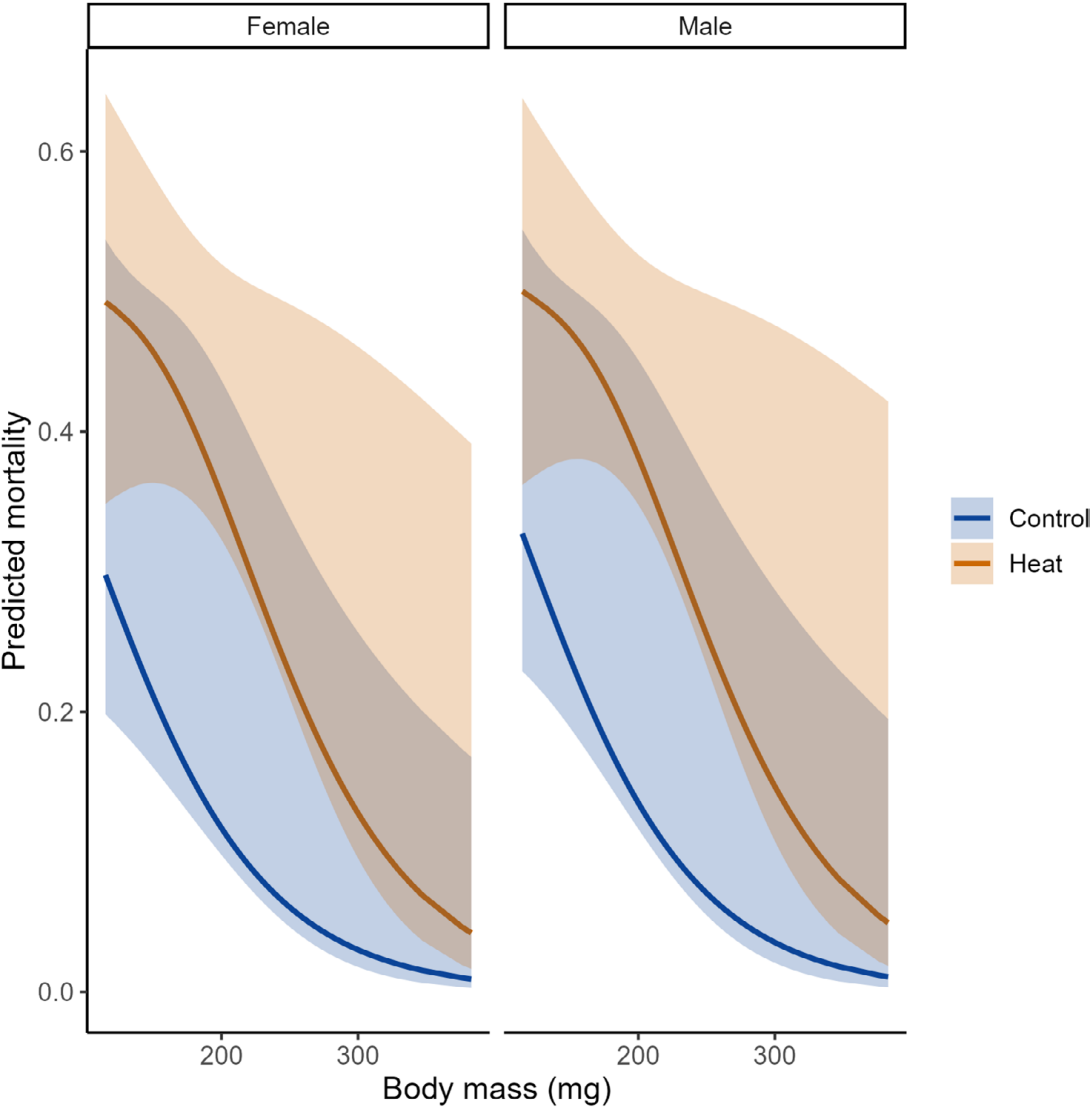
Predicted mortality (±95% confidence intervals) for males and females under control and heatwave treatments across body masses (shown in blue and orange, respectively). Lines and shaded areas represent predicted probabilities and 95% confidence intervals from a binomial generalised linear mixed model.

Body mass significantly influenced mortality rate in both sexes, with smaller males and females experiencing a higher mortality rate in both the control and heatwave treatments (LRχ^2^ = 8.04, df = 1, *p* = 0.005) (Figure 2). Body mass of experimental individuals did not differ between males and females (LRχ^2^ = 2.41, df = 1, *p* = 0.13) or between treatments (LRχ^2^ = 0.04, df = 1, *p* = 0.85) (Figure S1).

**FIGURE 3:**
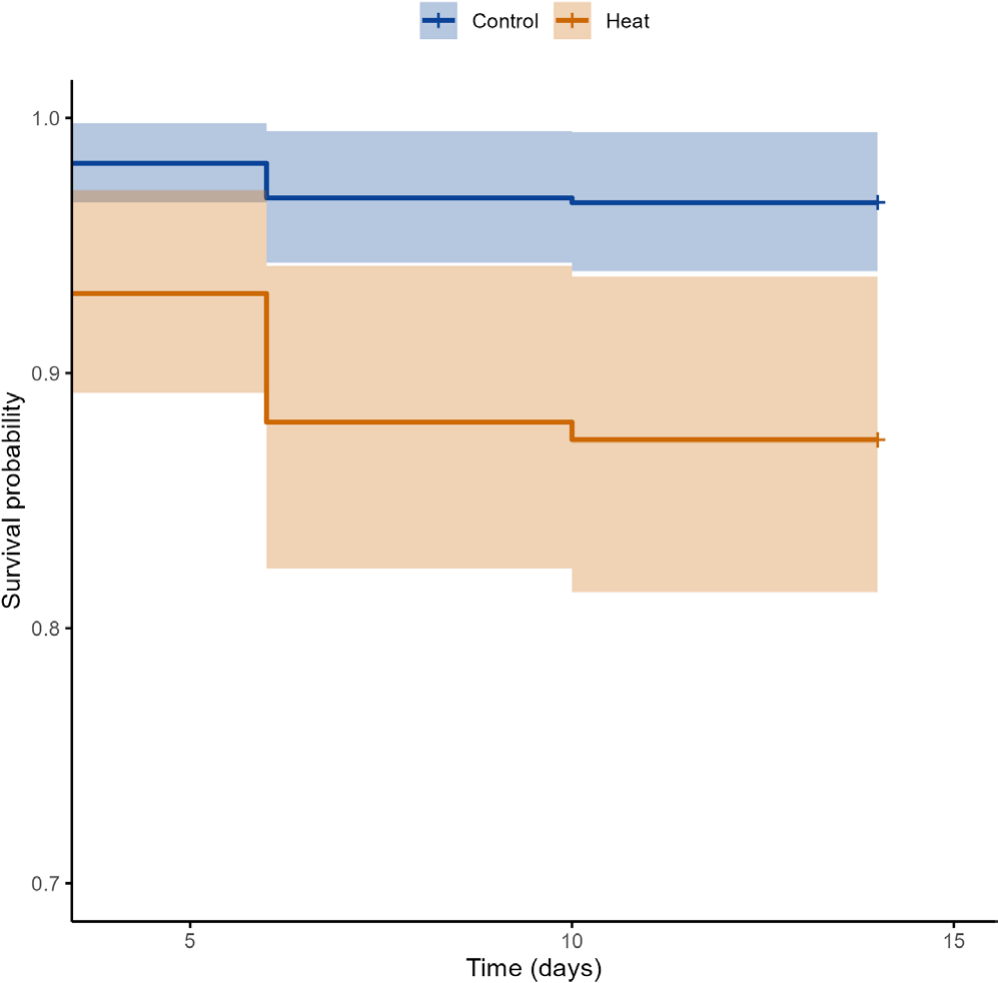
Predicted survival curves (±95% confidence intervals) from a Cox proportional hazards model comparing control and heat‐stress treatments (shown in blue and orange, respectively). Survival estimates begin immediately post‐heatwave. Curves are predicted holding body mass at the mean value and sex at the reference level (female). Curves are shown from the fixed‐effects model for visualisation.

Consistent with the previous results, the simulated heatwave also resulted in decreased survival over time, with a significantly higher likelihood of earlier mortality (HR = 4.03, 95% CI [1.84, 8.82], *p* < 0.001). There was no difference between the sexes (HR = 1.16, 95% CI [0.61, 2.21], *p* = 0.65). However, individuals with smaller body sizes were significantly more likely to die earlier, as the risk of death decreased at each interval by 1% for every milligramme of body mass (HR = 0.99, 95% CI [0.98, 1.00], *p* = 0.003) (Figure 3; Table 1).

## 4 Discussion

Understanding within‐population variation in response to temperature stress is vital for predicting population persistence in a warming world. Here, we exposed male and female burying beetles to an ecologically relevant simulated heatwave and found a substantial increase in mortality immediately following exposure to the heatwave. Despite our expectation, there were no sex differences in mortality either in the control or the heatwave treatment. Both males and females were over three times more likely to die following exposure to a heatwave compared to the control treatment. However, we found that smaller individuals had increased mortality in both control and heatwave treatments. Our findings highlight the consequences of heatwaves for mortality in both sexes and suggest that individuals with smaller body sizes are more vulnerable under heat stress, with possible implications for population demography.

This pattern of increased mortality under heat stress likely reflects underlying physiological trade‐offs. For instance, metabolic rate increases exponentially with temperature (Gillooly et al. 2001; Clarke and Fraser 2004; Lachenicht et al. 2010), which can increase mortality due to energy depletion (Storey and Storey 2004; Beuvard et al. 2022), or increased respiration introducing free radicals, causing oxidative stress (Zhang et al. 2023; Song et al. 2025). In addition, insect neural performance under prolonged or intense temperature stress will begin to affect muscular function, defined as the critical thermal maximum and induce a reversible coma before the insect dies (González‐Tokman et al. 2020). Indeed, heatwaves of ecological relevance have significant effects on mortality across a range of other insect taxa (e.g., McCauley et al. 2018; Moradinour et al. 2023; Cheng et al. 2025; Wiil and Pilakouta 2026). Global insect abundance and diversity are declining at an alarming rate, potentially driven by these effects of extreme heat stress events on insect mortality (e.g., Hallmann et al. 2017; Powney et al. 2019; Seibold et al. 2019). Given the importance of insects for ecosystem functioning (Weiskopf et al. 2020), and the particular importance of burying beetles for decomposition and nutrient cycling (Ilardi et al. 2021), our findings suggest considerable ecological implications.

Although we found an overall effect of heat stress on mortality, there was no evidence of sex‐specific effects. This is in line with a growing body of literature that has also found similar mortality rates across the sexes: in adult *Pieris napi* butterflies (Moradinour et al. 2023); adult *Rhizoglyphus robini* bulb mites (Parrett et al. 2024); in dragonfly larvae (*Erythemis collocate*; McCauley et al. 2018); and in four species of *Drosophila* (Mitchell and Hoffmann 2010). Interestingly, Grieve and Pilakouta (2026) found sex differences in temperature sensitivity of fertility in this same species. Similarly, equivalent lethal limits across the sexes but sex‐specificity in thermal sensitivity of fertility has been recorded in bulb mites (*R. robini* Parrett et al. 2024) and tsetse flies (*Glossina pallidipes*; Weaving et al. 2024). These findings suggest that sex differences in gametic functioning under thermal stress may be unrelated to wider sex differences in survival. Distinct mechanisms may underly each outcome, highlighting the need for caution when using results from one instance to predict the other.

One proposed explanation for divergent thermal tolerances between the sexes is differences in geographic range. Male mating strategies can lead to larger geographic ranges for mate searching (Lees et al. 2012; Todd and Nowakowski 2021; Yarwood et al. 2021), which could explain sex‐specific selection pressures for broader thermal limits in males (Pottier et al. 2021; Santini et al. 2025). However, *N. vespilloides* exhibit an unconventional breeding strategy that depends on fresh carrion for reproduction. As both males and females independently locate carrion to secure mating opportunities, reproductive success is closely tied to dispersal in both sexes. Mark–recapture studies of *N. americanus* found that individuals searching for carrion travelled an average of 1.23 km per night, with no difference between males and females (Creighton and Schnell 1998). These findings suggest that males and females likely occupy similar geographic ranges and experience comparable thermal environments, potentially resulting in similar thermal selection pressures across the sexes.

Furthermore, *N. vespilloides* are unique in that they do not strictly conform to traditional Darwin–Bateman sex roles. Ecological sex differences and sex‐specific heat sensitivity can arise from divergent evolutionary interests generating sexual conflict over reproductive rate and parental investment (Bonduriansky et al. 2008; Immonen et al. 2018; Tarka et al. 2018). In this species, both males and females provide parental care. Biparental care may reduce divergence in life‐history strategies when both sexes share a similar reproductive span and invest substantially in offspring (Hämäläinen et al. 2018). Such shared reproductive interests could constrain the evolution of strongly sex‐specific pace‐of‐life strategies. That said, even when providing care as a pair, females typically perform the majority of direct care, such as provisioning larvae (Engel et al. 2016; Pilakouta et al. 2018). Males are generally less consistent caregivers and may prioritise additional mating opportunities (Engel et al. 2016; Royle 2016; Potticary et al. 2024).

The absence of observed sex‐specificity in mortality under temperature stress could also be related to the absence of sexual size dimorphism in our study species. Sex differences in acclimation capacity were associated with the difference in body mass between females and males in a large meta‐analysis (Pottier et al. 2021). For example, in an insect species with sexual size dimorphism (mountain pine beetles, *Dendroctonus ponderosae* Hopkins), females had lower mortality compared to males, which was linked to body volume (Lachowsky and Reid 2014). The male and female beetles used in our experiment did not differ in body mass, which may partly explain the absence of sex‐specificity in heat sensitivity.

Indeed, body mass was associated with mortality in this species, providing support for the hypothesis that sexual size dimorphism may be linked to sex‐specific differences in heat sensitivity. We found that smaller body sizes in both male and female beetles were associated with significantly higher mortality rates across both control and heat treatments. Larger individuals tend to live longer and experience lower mortality rates across a range of taxa (e.g., Vadillo Gonzalez et al. 2021; Lorenzen 2022; Giménez and Jenkins 2024; Sentis et al. 2024). In ectotherms, smaller individuals may have lower energy reserves (Rall et al. 2012) and faster development times (Sentis et al. 2024). Fast development and maturation rates have been associated with lower individual quality and reduced longevity (Marty et al. 2011; Sentis et al. 2024). For example, early eclosion in the solitary parasitoid wasp *Aphaereta genevensis* resulted in a higher initial egg load but at the cost of reduced survival and longevity (Thorne et al. 2006).

Our findings also suggest that this increase in mortality in smaller individuals is maintained under stressful temperatures. Consistent with this pattern, lifespan under high temperatures decreased with smaller body size in the yellow dung fly *Scathophaga stercoraria* (Reim et al. 2006); larger Chilean pencil catfish *Trichomycterus areolatus* showed increased thermal tolerance than smaller individuals (Avilés‐Hernández et al. 2025); and larger *D. melanogaster* were more tolerant to heat stress than smaller individuals (Leiva et al. 2024). In contrast, comparative studies across ectothermic species often report that smaller‐bodied species exhibit greater tolerance to high temperatures than larger organisms (Leiva et al. 2019; Peralta‐Maraver and Rezende 2021), although this pattern is not universal (Antunes et al. 2024). This discrepancy highlights that interspecific patterns may not translate directly to intraspecific variation, which remains comparatively understudied.

Such size‐dependent effects on mortality could exacerbate the impact of climate change, if warming also drives a reduction in body size. For example, prior work in this study species has shown that heatwave exposure during early development on the carcass is associated with a lower larval mass, potentially as a result of decreased carcass quality under heat stress and reduced resource acquisition during the larval stage (Pilakouta et al. 2023, 2024). More broadly, declining body size has been posited as the third universal response to global warming, after distribution shifts and changes in phenology (Gardner et al. 2011). Warming‐induced body size reductions may arise due to underlying physiological mechanisms (Ohlberger 2013), such as oxygen or resource limitation during development (Verberk et al. 2021) or the adaptive value of a short development time (Ohlberger 2013). Thus, the predicted reduction in body size due to warming may be particularly detrimental across animal populations if it also results in a higher mortality rate.

In sum, we show that (i) heatwave exposure led to a significant increase in mortality rate compared to the control treatment, (ii) males and females suffered similar mortality both in the control and heatwave treatments and (iii) a smaller body size was associated with increased mortality across both treatments. The absence of sex‐specific effects of thermal stress on mortality, despite evidence for sex‐specificity in thermal sensitivity of fertility in this species, suggests distinct underlying mechanisms. Thus, differences between males and females in their vulnerability to thermal stress in terms of fertility do not necessarily translate into corresponding differences in survival. In addition, our findings of size‐dependent effects suggest that warming‐induced reductions in body size may also indirectly result in higher mortality rates, thereby exacerbating the effects of climate change. This work therefore provides new insights into the potential roles of sex and body size in influencing the vulnerability of organisms to warming and highlights the need to better understand such sources of intraspecific variation in mortality under heat stress. More broadly, our findings add to growing evidence that extreme climatic events represent a major challenge for insects. As heatwaves become more frequent and severe, differential mortality among individuals may contribute to changes in population size and patterns of intraspecific variation, ultimately affecting the persistence of insect populations in a warming world.

## Author Contributions

**Isobel Grieve:** conceptualization (equal), data curation (lead), formal analysis (lead), methodology (equal), visualization (lead), writing – original draft (lead). **Natalie Pilakouta:** conceptualization (equal), funding acquisition (lead), methodology (equal), resources (lead), supervision (lead), writing – review and editing (lead).

## Funding

The study was funded by a Royal Society Research Grant awarded to NP (RGS\R1\211295).

## Conflicts of Interest

The authors declare no conflicts of interest.

## Supporting information


**Data S1:** Data.

**Data S2:** R code.

**Figure S1:** Body mass (mg) of female and male individuals. Boxplots display the median, interquartile range and 1.5 × IQR whiskers, with jittered points representing individual beetles.

## Data Availability

All relevant data are available on the Dryad Digital Repository: https://doi.org/10.5061/dryad.d7wm37qgx (Grieve and Pilakouta 2026).
