# Supplementary figures and images for: Thermal Stress Has Size‐Dependent but Not Sex‐Specific Effects on Mortality in an Insect Model
Source: Ecol Evol. 2026 Jul 12;16(7):e74020. doi: 10.1002/ece3.74020 (PMC13357381; doi:10.1002/ece3.74020)

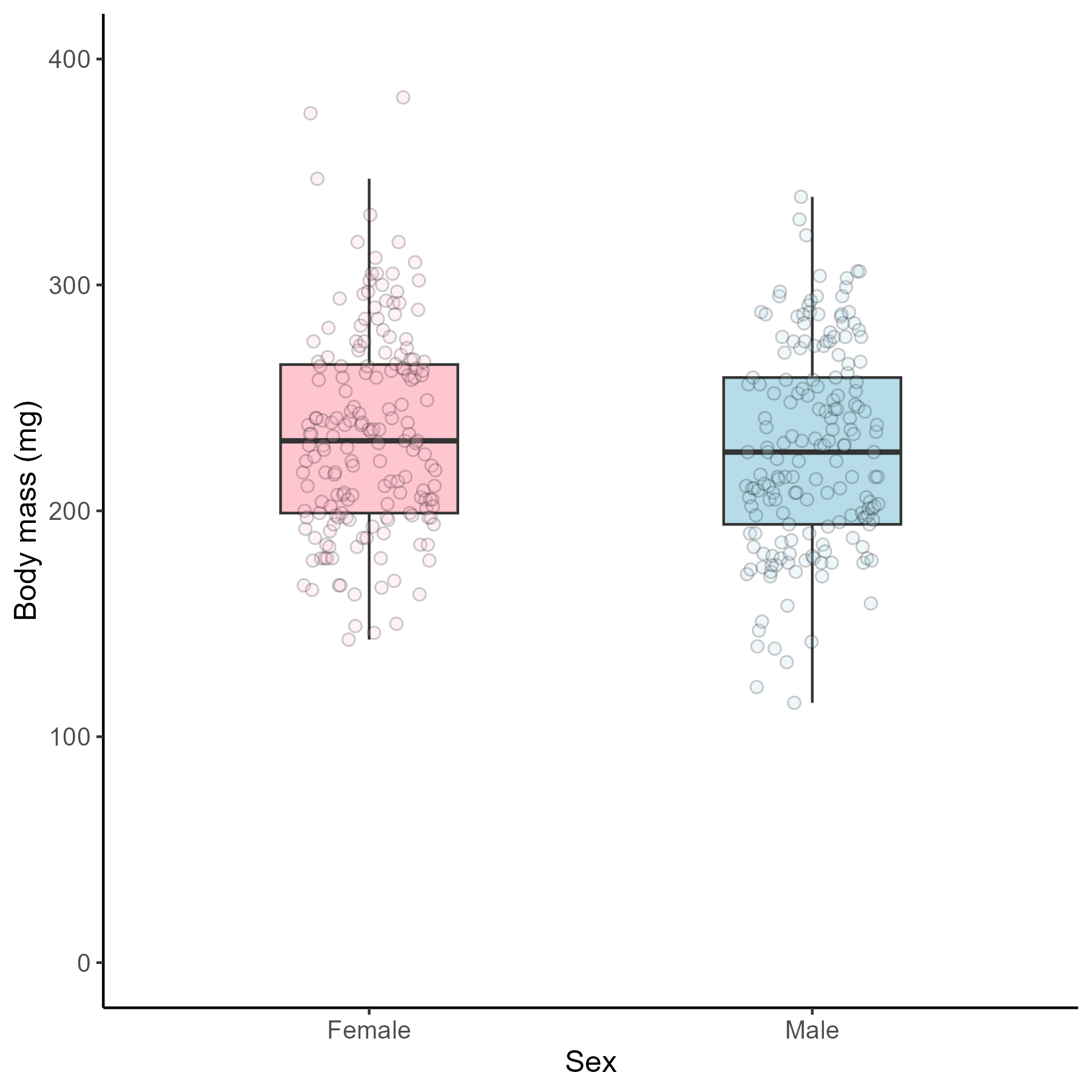

Supplement: Supplementary file 3 — Figure S1: Body mass (mg) of female and male individuals. Boxplots display the median, interquartile range and 1.5 × IQR whiskers, with jittered points representing individual beetles. [file ECE3-16-e74020-s003.png]
